# *Saccharomyces cerevisiae* Cells Lacking the Zinc Vacuolar Transporter Zrt3 Display Improved Ethanol Productivity in Lignocellulosic Hydrolysates

**DOI:** 10.3390/jof8010078

**Published:** 2022-01-14

**Authors:** Joana Terra-Matos, Marta Oliveira Teixeira, Cátia Santos-Pereira, Henrique Noronha, Lucília Domingues, Carmen Sieiro, Hernâni Gerós, Susana Rodrigues Chaves, Maria João Sousa, Manuela Côrte-Real

**Affiliations:** 1 Centre of Molecular and Environmental Biology (CBMA), Department of Biology, Campus de Gualtar, University do Minho, 4710-057 Braga, Portugal; joana_matos.5@hotmail.com (J.T.-M.); martasofia.teixeira@hotmail.com (M.O.T.); catia91pereira@gmail.com (C.S.-P.); henriquelsnoronha@gmail.com (H.N.); geros@bio.uminho.pt (H.G.); suchaves@bio.uminho.pt (S.R.C.); mjsousa@bio.uminho.pt (M.J.S.); 2 Centre for Textile Science and Technology (2C2T), Department of Textile Engineering, Campus of Azurém, University of Minho, 4800-058 Guimarães, Portugal; 3 Centre of Biological Engineering (CEB), Department of Biological Engineering, University of Minho, 4710-057 Braga, Portugal; luciliad@deb.uminho.pt; 4 Centre for the Research and Technology of Agro-Environmental and Biological Sciences (CITAB), University of Trás-os-Montes and Alto Douro, 5001-801 Vila Real, Portugal; 5 Biomedical Research Center (CINBIO), Department of Functional Biology and Health Sciences, Faculty of Biology, University of Vigo, 36310 Vigo, Spain; mcsieiro@uvigo.es

**Keywords:** *Saccharomyces cerevisiae*, acetic acid, lignocellulosic hydrolysates, vacuolar zinc transporter, lignocellulosic ethanol

## Abstract

Yeast-based bioethanol production from lignocellulosic hydrolysates (LH) is an attractive and sustainable alternative for biofuel production. However, the presence of acetic acid (AA) in LH is still a major problem. Indeed, above certain concentrations, AA inhibits yeast fermentation and triggers a regulated cell death (RCD) process mediated by the mitochondria and vacuole. Understanding the mechanisms involved in AA-induced RCD (AA-RCD) may thus help select robust fermentative yeast strains, providing novel insights to improve lignocellulosic ethanol (LE) production. Herein, we hypothesized that zinc vacuolar transporters are involved in vacuole-mediated AA-RCD, since zinc enhances ethanol production and zinc-dependent catalase and superoxide dismutase protect from AA-RCD. In this work, zinc limitation sensitized wild-type cells to AA-RCD, while zinc supplementation resulted in a small protective effect. Cells lacking the vacuolar zinc transporter Zrt3 were highly resistant to AA-RCD, exhibiting reduced vacuolar dysfunction. Moreover, *zrt3*Δ cells displayed higher ethanol productivity than their wild-type counterparts, both when cultivated in rich medium with AA (0.29 g L^−1^ h^−1^ versus 0.11 g L^−1^ h^−1^) and in an LH (0.73 g L^−1^ h^−1^ versus 0.55 g L^−1^ h^−1^). Overall, the deletion of *ZRT3* emerges as a promising strategy to increase strain robustness in LE industrial production.

## 1. Introduction

Lignocellulosic ethanol (LE) has gained increasing public attention as a second-generation biofuel. Indeed, it produces less greenhouse gases (GHGs) and can be obtained from inexpensive and abundant agricultural and forestry residues, and thus its production does not require the food resources that first-generation biofuels do [1,2]. In addition to the positive impact from an environmental perspective, the use of biomass over fossil fuels presents unquestionable advantages from economic and health standpoints [3]. Yeasts, in particular engineered *Saccharomyces cerevisiae* strains with improved tolerance to lignocellulosic hydrolysate inhibitors, have been the microorganisms most commonly explored for bioethanol production from LH (Table 1) [4]. However, lignocellulosic hydrolysis-derived inhibitors, such as weak acids (e.g., acetic, formic and levulinic acids), furan aldehydes (e.g., furfural and 5-hydroxymethylfurfural (HMF)), and phenolic compounds, together with increasing concentrations of ethanol along fermentation, cause severe stress that limits ethanol productivity [5,6]. Improving the resistance of yeast cells to such different stresses will thus boost fermentative efficiency [7] and determine LE production sustainability.

Acetic acid (AA) is usually the most abundant lignocellulosic hydrolysis-derived inhibitor [17]. Above a certain concentration threshold, it inhibits yeast cell growth by hindering metabolic functions through intracellular accumulation and acidification [18,19,20,21]. At high concentrations, it induces a mitochondrial-mediated regulated cell death (RCD) process with features similar to mammalian apoptosis [22,23], namely, reactive oxygen species (ROS) accumulation [23,24]. AA-induced RCD (AA-RCD) also involves a partial permeabilization of the vacuolar membrane and the release of the Pep4 protease to the cytosol while inhibiting autophagy [25]. A subset of yeast strains lacking vacuolar proteins were found to be resistant to AA in a genome-wide screen, highlighting the executing role of the vacuole in cell demise induced by this compound [26]. 

It has been suggested that superoxide dismutase (SOD) activity can increase transiently in cells undergoing AA-RCD, as an adaptation mechanism to cope with superoxide anion accumulation, and that catalase (CAT) activity is important to protect cells from AA-RCD [27]. Cytosolic antioxidant enzymes Sod1 and catalase T have zinc as a cofactor, and thus zinc plays an important role in yeast stress resistance, including ethanol and acid stress [28]. Indeed, among other protein-protective effects against oxidative stress, zinc increases the activity of catalases and superoxide dismutases, consequently reducing the deleterious effects of oxidant-promoting enzymes [29]. Accordingly, zinc sulphate addition has been shown to increase cell viability and ethanol production during high gravity ethanol fermentation [30]. Improved growth and ethanol productivity under AA stress by zinc supplementation was also observed [28,31,32]. The availability of zinc in the cytosol is regulated by specific transporters, both at the plasma (Zrt1 and Zrt2) and vacuolar membrane (Zrt3, Cot1 and Zrc1). Zrt3 mediates the efflux of stored zinc from the vacuole to the cytosol and its expression is upregulated under low zinc conditions, like that of Zrt1 and Zrt2 [33,34,35]. On the other hand, Cot1 and Zrc1 mediate vacuolar zinc accumulation under zinc-replete conditions. Under these conditions, the *zrt3*Δ strain accumulates zinc at higher levels than the wild-type strain by upregulating zinc uptake, even though it is unable to mobilize vacuolar zinc stores [36].

Given the bottlenecks and challenges still faced by the biotechnological industry of yeast-based bioethanol production from lignocellulosic biomass [4], a deeper understanding of AA-RCD may provide new clues towards the improvement of fermentative strategies. Because previous studies have shown that both vacuolar proteins and zinc play a role in yeast AA-RCD, the current study sought to address the involvement of the zinc vacuolar transporter Zrt3 in this process. It was observed that the Zrt3 transporter is involved in the execution of cell demise in response to AA, and that its absence improves cell growth and ethanol productivity. The *zrt3*Δ mutant therefore emerges as a more advantageous and cost-effective alternative for sustainable industrial LE production than LH supplementation with zinc. 

## 2. Materials and Methods

### 2.1. Yeast Strains

*Saccharomyces cerevisiae* (*S. cerevisiae*) strains used in this work: BY4741 wild-type (MATa his3Δ1 leu2Δ0 met15Δ0 ura3Δ0) and *zrt3*Δ (BY4741 *ZRT3::kanMX4)*, purchased from EUROSCARF. For fluorescence microscopy studies, strains BY4741 and *zrt3*Δ transformed with pRS413-Pep4-mCherry (HIS3) were used.

### 2.2. Media and Growth Conditions

*S. cerevisiae* strains were cultivated on YEPD (Yeast Extract Peptone Dextrose) medium plates (1% (*w*/*v*) yeast extract, 2% (*w*/*v*) bactopeptone, 2% (*w*/*v*) glucose and 2% (*w*/*v*) agar) at 30 °C for 2 d. Cells were then transferred to liquid YEPD and cell density was adjusted to an OD_640 nm_ of 0.0005–0.005 (depending on the strain), and incubated at 30 °C with 200 rpm agitation until the mid-exponential phase (OD_640 nm_ of 0.5) before initiating the different assays performed throughout this work.

Cells that were transformed with the pRS413 plasmid were selected and cultivated in synthetic complete medium without histidine (2% (*w*/*v*) glucose; 0.5% (*w*/*v*) ammonium sulphate; 0.7% (*w*/*v*) yeast nitrogen base w/o ammonium sulphate and w/o amino acids; 0.2% (*w*/*v*) dropout mixture; 0.01% (*w*/*v*) uracil and tryptophan; 0.02% (*w*/*v*) leucine).

### 2.3. Cell Viability Assays

Yeast cells were cultivated as stated above (Section 2.2). At the exponential growth phase, cells were collected and resuspended in the appropriate medium adjusted to pH 3.0. The treatment was carried out by adding AA to the resuspended cells, followed by incubation at 30 °C under agitation at 200 rpm. Cells were collected 120 min after addition of AA and 50 μL of culture at OD_640 nm_ = 1 was diluted in a total of 1 mL of sterile deionized water, followed by 10^−4^ serial dilutions. Five 40 μL drops were withdrawn from the 10^−4^ dilution and plated in YEPD plates, grown for 2 d at 30 °C, and cell viability was assessed by counting the Colony Forming Units (c.f.u.). The percentage of cell survival was calculated from the ratio between the mean number of counted colonies over the mean number of colonies at time zero of the respective strain, multiplied by 100.

### 2.4. Assessment of Reactive Oxygen Species (ROS) Accumulation in Response to Acetic Acid Treatment by Flow Cytometry

Total ROS production was assessed with the superoxide anion indicator dihydroethidium (DHE) (Molecular Probes, Eugene, OR, USA). In the absence of superoxide anion, this probe exhibits blue fluorescence in the cytosol, but, when oxidized by the anion, it intercalates into nucleic acids displaying red fluorescence in the nucleus. The fluorescence probe Mitosox (Life Technologies, Carlsbad, CA, USA) was used to detect mitochondrial ROS production. It is a live-cell permeant dye that rapidly and selectively targets the mitochondria, where it is exhibits red fluorescence after oxidation by superoxide anion [37].

Cells were treated with AA as described above (Section 2.2), collected by centrifugation at 3000× *g* for 3 min, washed with deionized water and resuspended in 500 µL of PBS. Cells were then stained with DHE and Mitosox at a final concentration of 5 µg/mL and 1.5 µg/mL, for 20 and 45 min at room temperature, respectively. After staining, the samples were analyzed by flow cytometry.

Flow cytometry assays were carried out using an Epics^®^ XL™ (Beckman Coulter, Brea, CA, USA) flow cytometer, equipped with an argon ion laser emitting a 488 beam. Fluorescence was collected by the FL-3 (605–635 nm) and FL-4 (660–700 nm). For each sample, 30,000 events were acquired and processed using the FlowJo^®^ 7.6 software (Becton, Dickinson & Company, Franklin Lakes, NJ, USA).

### 2.5. Assessment of Catalase and Superoxide Dismutase Activity

#### 2.5.1. Preparation of Protein Extracts

To prepare total protein extracts, cells were cultivated and treated as described above. After treatment, cells were collected by centrifugation at 3000× *g* for 3 min and washed with 20 mL of H_2_O. Cell lysis was carried out in a FASTprep equipment (5–10 × 30 s cycles) in lysis buffer (0.6 M Mannitol, 2 mM EGTA, 10 mM Tris, 1 protease inhibitor cocktail tablet without EDTA/10 mL, pH 6.8) containing glass beads (1/3 volume). Cell disruption was checked under the optical microscope. Cell debris was removed by centrifugation at 13,000× *g* for 10 min at 4 °C, and the supernatant was collected. Protein concentration was determined by the Lowry method [38].

#### 2.5.2. Assessment of Mitochondrial Superoxide Dismutase Activity by Native Gel Electrophoresis 

Protein extracts (10, 20 or 60 μg) were separated by 15% non-denaturing polyacrylamide gel electrophoresis (native-PAGE) to assess Sod1 activity. Electrophoresis was run for approximately 3 h at 20 mA per gel at 4 °C. In each assay, samples were run in duplicate to perform parallel staining with Coomassie blue (Fisher Scientific, Hampton, NH, USA) and Nitro Blue Tetrazolium (NBT) (Roche Molecular Systems Inc., Basel, CH). To this end, one of the gels was stained with a solution containing 50 mM potassium phosphate (pH 7.8), 0.1 μL/mL TEMED, 0.13 mg/mL NBT, and 0.1 mg/mL riboflavin for 45 min in the dark. After 45 min of incubation under slow agitation, the gel was exposed to light for 2 min. Under light exposure, riboflavin is reduced, which leads to the production of superoxide radicals. Then, O^2−^ is reduced by NBT to create insoluble blue formazan. Sod1 activity is visualized as a colourless band in the blue background because Sod1 scavenges the superoxide, thus inhibiting the blue colour formation [39]. The second gel was carefully transferred to a Coomassie staining solution (0.2% Coomassie blue, 7.5% of AA, 40% ethanol) for 20 min under stirring. Subsequently, the staining solution was removed, and the gel was washed with de-staining solution (10% of AA, 20% ethanol) several times until the blue bands were observed.

Photos of both gels were taken with a transilluminator, quantification performed using ImageJ Fiji software (ImageJ, Bethesda, MD, USA) and NBT normalized to Coomassie blue.

#### 2.5.3. Assessment of Catalase Activity

Catalase activity was measured with a Clark electrode (Kipp & Zonen, Delft, NL, USA) to monitor the rate of O_2_ production. The final protein concentration of each sample was set to 0.03 μg/μL in 2 mL of 50 mM phosphate buffer (pH 7.0). After obtaining a baseline, the reaction was started with the addition of 0.75 mM H_2_O_2_. This process was repeated 3 times for each sample/condition.

### 2.6. Assessment of Pep4 Localization and Vacuolar Membrane Permeabilization by Fluorescence Microscopy

The wild-type and *zrt3*Δ strains expressing Pep4-mCherry were treated as described above (Section 2.2). To assess vacuolar membrane permeabilization (VMP), cells were collected and stained for 20 min in the dark with Celltracker™ Blue CMAC (7-amino-4-chloromethylcoumarin) (Molecular Probes, Eugene, OR, USA) at a final concentration of 2 µM, using a stock solution of 100 µM in Dimethyl sulfoxide (DMSO). Under normal conditions, this probe is sequestered in the vacuole exhibiting a bright blue fluorescence. However, when the vacuolar membrane is compromised, it displays a diffuse pattern [40]. This procedure was used to assess Pep4 localization and VMP during AA treatment.

Fluorescence microscopy experiments were performed with a Leica Microsystems DM-5000B microscope (Leica Microsystems, Wetzlar, DE, Germany), with the appropriate filter settings (DIC (differential interference contrast), red and blue) with a 100× objective. Images were obtained with a Leica DFC350 FX Digital Camera (Leica, Wetzlar, DE, Germany) and processed with LAS AF Microsystems software (Leica, Wetzlar, DE, Germany). For phenotype quantification, at least 300 cells were counted in three independent experiments.

### 2.7. Evaluation of Vacuolar pH by Flow Cytometry

The fluorescent probe 5-(and -6)-carboxy-2′, 7′-dichlorofluorescein diacetate (CDCFDA) (Sigma-Aldrich, St. Louis, MO, USA) was used to analyze vacuolar pH variations in AA-treated cells. This probe accumulates in acidic organelles and undergoes pH-dependent changes, producing a more intense fluorescence as vacuoles become more alkaline [41,42]. A stock of 1 mM of CDCFDA was prepared in DMSO and kept at −20 °C.

Cells were treated with AA as described above (Section 2.2), collected (1 mL at OD_640 nm_ of 0.1), washed with deionized water and resuspended in 1 mL of CF buffer (50 mM glycine, 10 mM NaCl, 5 mM KCl, 1 mM MgCl_2_, 40 mM Tris, 100 mM MES (2-(N-morpholino) ethanesulfonic acid), 2% (*v*/*w*) glucose, pH 4.5). The probe was added to a final concentration of 1.6 µM and the mixture was incubated at 30 °C for 20 min, at 200 rpm. Cells were collected, washed with CF buffer without glucose supplementation, resuspended in 700 µL of the same buffer, and analyzed by flow cytometry [43].

Data acquisition was performed in the same equipment as in Section 2.4 and fluorescence was collected using FL-1 (505–545 nm).

### 2.8. Zinc Supplementation and Limitation Assays

For zinc supplementation assays, cells cultivated and resuspended as described above were treated with 10 mM ZnSO_4_, 100 mM AA, and with 10 mM ZnSO_4_ plus 100 mM AA, and incubated at 30 °C, 200 rpm. Cells were harvested before (0 min) and 60 min after the addition of AA and/or ZnSO_4_ to assess cell viability and cellular accumulation of superoxide anion. As a control, cells were processed under the same conditions but in the absence of both ZnSO_4_ and AA.

For zinc limitation assays, cells were pre-treated with and without 1 mM EDTA (stock solution of 100 mM), for 30 min at 30 °C and 200 rpm. Then, the cells cultivated and resuspended as stated above were treated with 1 mM EDTA plus 75 mM AA, and incubated at 30 °C, 200 rpm. Cells were harvested before (0 min) and 90 min after the addition of AA and EDTA to assess cell viability and cellular accumulation of superoxide anion. Controls were prepared in the absence of EDTA and AA.

### 2.9. Assessment of zrt3*Δ* Cell Growth and Fermentative Performance in the Presence of Acetic Acid

To assess cell growth of wild-type and *zrt3*Δ strains, cells were pre-inoculated on YEPD, at 30 °C and 200 rpm, overnight. The culture was then set at an OD_640 nm_ of 0.5 in YEPD medium containing 40 g/L glucose, pH 4.5, before addition of 60 mM AA. Controls were performed in the absence of AA. Cell growth was evaluated by measuring the OD_640 nm_ along 32 h. Lag phase time was determined as the time required to double the initial optical density [44]. Glucose and ethanol in the medium were measured by HPLC (Beckman Coulter, Brea, CA, USA) [45].

### 2.10. Assessment of zrt3*Δ* Cell Growth and Fermentative Performance in a Lignocellulosic Hydrolysate 

Wild-type and *zrt3*Δ cells were cultivated as described in Section 2.9, but in a different medium. Vine pruning residue (Vpr) was submitted to hydrothermal treatment (autohydrolysis) under non-isothermal conditions (Tmax of 220 °C) in a 2 L stainless steel reactor (Parr Instruments Company, Moline, IL, USA) equipped with Parr PDI temperature controller (model 4848) at liquid to solid ratio of 6 g distilled water/1 g of Vpr. After autohydrolysis, solid and liquid phases were separated by filtration and autohydrolysis liquors (liquid phase) were supplemented with 1% (*w*/*v*) yeast extract, 2% (*w*/*v*) bactopeptone, 4% (*w*/*v*) glucose, and adjusted to pH 4.5 with NaOH and used as culture medium for growth assays. An aliquot of autohydrolysis liquors (liquid phase) was filtered through 0.45 μm membranes and used for direct HPLC determination of AA, formic acid, hydroxymethylfurfural (HMF) and furfural as described above. Cell growth was evaluated by measuring the OD_640 nm_ for 30 h. Lag phase time was determined as described in Section 2.9.

## 3. Results

### 3.1. The zrt3*Δ* Strain Is Resistant to Acetic Acid-Induced Cell Death

**Figure 1:**
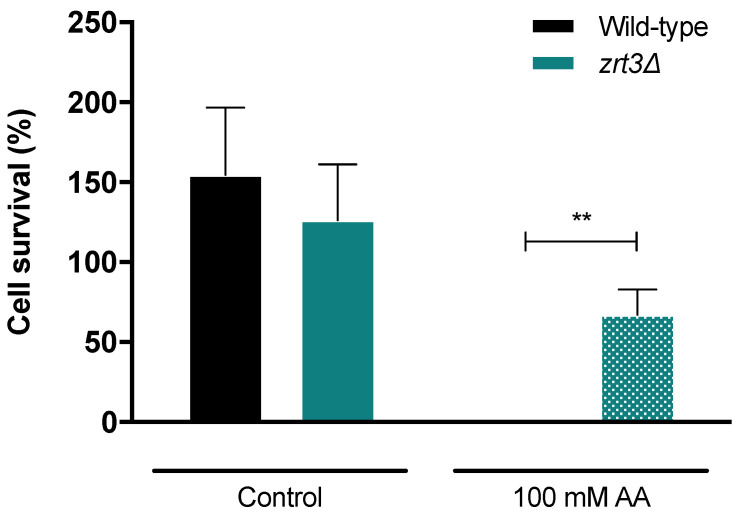
Cell survival of wild-type and *zrt3*Δ cells after AA treatment. Cell survival was assessed by c.f.u. 120 min after treatment with 100 mM AA, pH 3.0. Values are mean ± SEM (*n* ≥ 3). Statistical analysis was performed by two-way ANOVA. ** *p* < 0.01 in comparison with the wild-type strain.

Cell survival assays showed that *ZRT3* deletion increases resistance of cells to AA treatment (100 mM, pH 3.0) in comparison with the wild-type strain. Indeed, cell survival of wild-type cells after 120 min of treatment was close to zero, while most *zrt3*Δ cells remained alive (ca. 70% survival) (Figure 1).

### 3.2. The Acetic Acid Resistance Phenotype of zrt3*Δ* Is Associated with Lower Accumulation of Superoxide Anion and Increased Activity of Catalases

**Figure 2:**
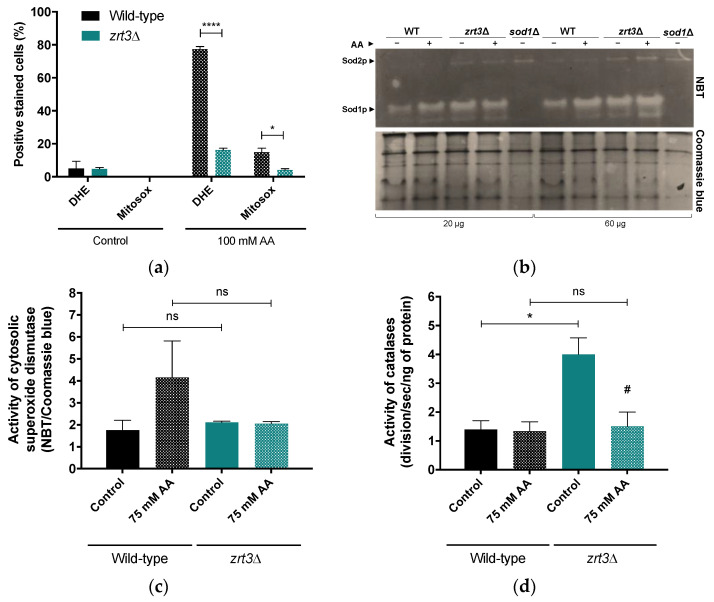
The effect of AA on superoxide anion accumulation and Sod1 and catalase enzymatic activities in wild-type and *zrt3*Δ cells. (**a**) Cellular and mitochondrial superoxide anion accumulation in *S. cerevisiae* BY4741 wild-type and *zrt3*Δ strains 60 min after incubation with AA (100 mM), pH 3.0 or in the absence of AA. Values represent the percentage of cells with positive DHE and Mitosox staining and are the mean ± SEM (*n* = 2). Statistical analysis was performed by two-way ANOVA. * *p* < 0.05, **** *p* ˂ 0.0001 in comparison with the wild-type strain. (**b**) To evaluate Sod1 activity in WT and *zrt3*Δ mutant 60 min after incubation with AA (75 mM) or in the absence of AA, protein extracts were run on native polyacrylamide gels (15%) and stained with NBT and Coomassie blue. Two different concentrations of each condition (20 and 60 µg) are shown. As a Sod1 control, the *sod1*Δ mutant was used. Representative gels of these assays are shown. (**c**) To measure enzymatic activity, the intensity of gel bands from (**b**) was quantified using Image J software. Values are the mean ± SEM (*n* = 3). Statistical analysis was performed by one-way ANOVA. Non-significant values are represented as ns. (**d**) Determination of activity of catalases was estimated by measuring oxygen consumption with a Clark electrode. Activity of catalases was evaluated in 3 replicates of the same sample using 0.75 mM H_2_O_2_. Values are the mean ± SEM (*n* = 3). Statistical analysis was performed by one-way ANOVA * *p* < 0.05, in comparison with the wild-type strain. # *p* < 0.05 in comparison with the control.

To address whether the AA resistance phenotype of *zrt3*Δ was associated with lower mitochondrial ROS accumulation, cellular and mitochondrial superoxide anion accumulation was measured by flow cytometry after staining with DHE and Mitosox, respectively. Both cellular and mitochondrial superoxide anion accumulation in response to AA decreased significantly in the *zrt3*Δ mutant, as only less than 20% and 5% of the cells were stained with DHE and Mitosox, respectively. In contrast, the wild-type strain exhibited around 80% and 15% of DHE- and Mitosox-positive cells, respectively, (Figure 2a). Therefore, the next step was to evaluate how the activities of superoxide dismutases and catalases are affected by *ZRT3* deletion, which is associated with increased cellular zinc levels [36]. *zrt3*Δ cells displayed a significantly higher basal activity of catalases than wild-type cells (Figure 2d), while Sod1 activity was not significantly different (Figure 2b,c). Moreover, AA did not affect the activity of catalases in the wild-type strain, but significantly decreased the enhanced basal activity of catalases in *zrt3*Δ cells (Figure 2d). On the other hand, treatment with AA increased the Sod1 activity of the wild-type strain, though this was not statistically significant, while it had no effect in *zrt3*Δ cells (Figure 2c). 

### 3.3. The zrt3*Δ* Acetic Acid Resistance Phenotype Is Associated with Delayed Vacuolar Alterations and Maintenance of Vacuolar pH

**Figure 3:**
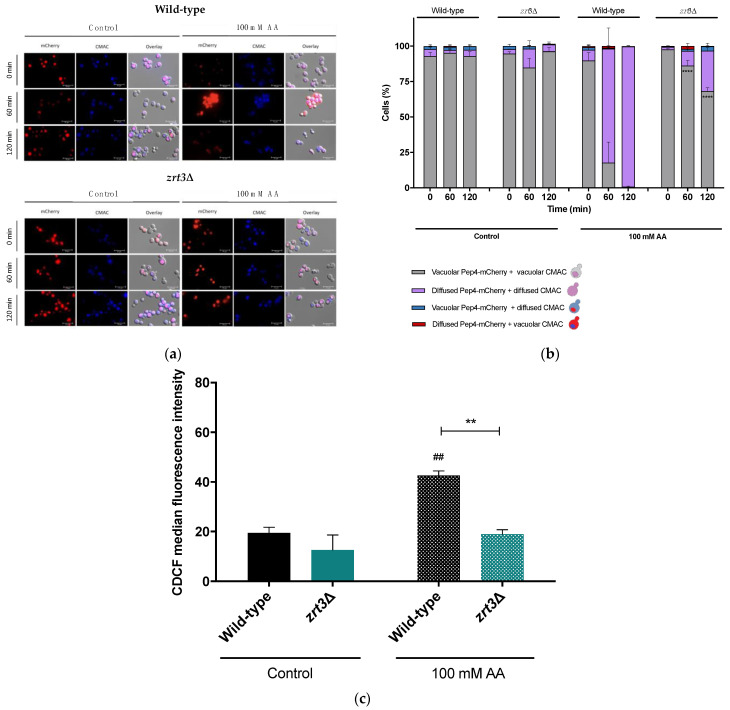
The effect of AA on Pep4-mCherry translocation from the vacuole into the cytosol, vacuolar membrane permeabilization and vacuolar pH in *S. cerevisiae* wild-type and *zrt3*Δ strains treated with AA. (**a**) Representative fluorescence microscopy images of wild-type and *zrt3*Δ cells transformed with Pep4-mCherry. Cells were incubated up to 120 min with or without AA and then stained with 2 µM CMAC to assess vacuole membrane integrity. Cells were observed under the fluorescence microscope with a 100× oil immersion objective. (**b**) Quantification of Pep4-mCherry localization and cells displaying VMP. At least 300 cells were counted for each condition. Values are the mean ± SEM (*n* = 3). Statistical analysis was performed by two-way ANOVA. **** *p* < 0.0001 in comparison with AA-treated wild-type cells. (**c**) Quantification of CDCF median fluorescence intensity of *S. cerevisiae* BY4741 wild-type and *zrt3*Δ cells 60 min after AA treatment (100 mM), pH 3.0. Cells were stained with 1.6 µM CDCFDA and analyzed by flow cytometry. Values are the median ± SEM (*n* = 3). Statistical analysis was performed by two-way ANOVA. ^##^ *p* < 0.01 in comparison with the control of wild-type cells; ** *p* < 0.01 in comparison with the AA-treated wild-type strain.

To further explore the role of Zrt3 in vacuolar dysfunctions occurring in the cell death process under study, the *zrt3*Δ strain was transformed with pRS413-Pep4-mCherry to monitor Pep4 localization in response to AA by fluorescence microscopy. About 80% of the wild-type cells exhibited Pep4-mCherry translocation from the vacuole to the cytosol 60 min after AA treatment, whereas Pep4-mCherry translocation was observed only in ca. 15% of *zrt3*Δ cells. This difference was equally apparent after 120 min (Figure 3a,b). To evaluate vacuolar membrane permeabilization (VMP), cells transformed with pRS413-Pep4-mCherry were stained with the vacuolar dye CMAC, as reported in Materials and Methods. As shown in Figure 3a, the majority of the wild-type cells displayed concomitant Pep4 release and VMP both 60 and 120 min after AA treatment. In contrast, only ca. 15% of *zrt3*Δ mutant cells exhibited red and blue fluorescence diffused into the cytosol 60 min after AA treatment, indicating a significant delay/decrease in VMP and Pep4 release. In parallel, the results show that AA induced a 2.0-fold increase in CDCF fluorescence, indicating an increase in vacuolar pH in the wild-type cells, whereas it did not affect the vacuolar pH of *zrt3*Δ cells (Figure 3c). These data are in accordance with the delayed VMP observed in AA-treated *zrt3*Δ cells.

### 3.4. Loss of Cell Survival and Superoxide Anion Accumulation Induced by Acetic Acid Are Affected by Zinc Availability

**Figure 4:**
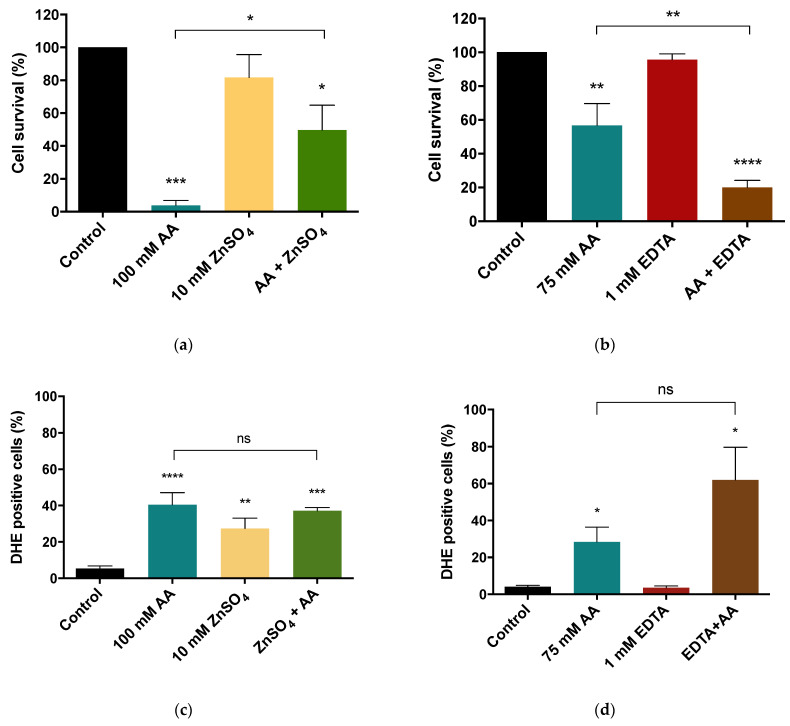
Effect of zinc supplementation (**a**) or limitation (**b**) on AA-induced cell death and cellular superoxide anion accumulation (**c**,**d**) in *S. cerevisiae* BY4741 wild-type cells. (**a**) Cells were treated with 10 mM ZnSO_4_, 100 mM AA or co-treated with both for 60 min. (**b**) Cells were pre-treated with and without 1 mM EDTA for 30 min and next treated with 1 mM of EDTA, 75 mM of AA or co-treated with both for 90 min. Cell survival was assessed by c.f.u. counting. (**c**) Quantification of the percentage of DHE-positive stained cells after supplementation with 10 mM ZnSO_4_, 100 mM AA or both for 60 min. (**d**) Quantification of the percentage of cells with DHE-positive staining after pre-treatment with and without 1 mM EDTA for 30 min, followed by treatment with 1 mM of EDTA, 75 mM of AA or both for 90 min. Values are percentages of DHE-positive cells ± SEM of three (**a**,**b**), five (**c**) and three (**d**) independent experiments. Statistical analysis was performed by one-way ANOVA. * *p* < 0.05, ** *p* < 0.01, *** *p* < 0.001, **** *p* ˂ 0.0001 in comparison with the control, or between indicated conditions. Non-significant values are represented as ns.

ZnSO_4_ increased the cell survival in response to AA from 10 to 50% (Figure 4a), indicating that supplementation with ZnSO_4_ protects cells from AA-RCD. In agreement, limiting zinc (in the presence of EDTA) decreased cell survival (Figure 4b). In experiments with zinc limitation, 75 mM of AA was used instead of 100 mM (used in zinc supplementation), since co-treatment with EDTA and 100 mM AA was extremely toxic.

To uncover whether ROS accumulation is involved in the effect of zinc availability on AA-RCD, cellular accumulation of superoxide anion was evaluated under zinc supplementation and limitation conditions by flow cytometry using the DHE probe. Zinc supplementation did not alter AA-induced ROS accumulation (Figure 4c), while zinc limitation (in the presence of EDTA) promoted an apparent increase in DHE-positive cells, but that was not statistically significant.

### 3.5. zrt3*Δ* Displays Improved Fermentative Performance in the Presence of Acetic Acid

**Figure 5:**
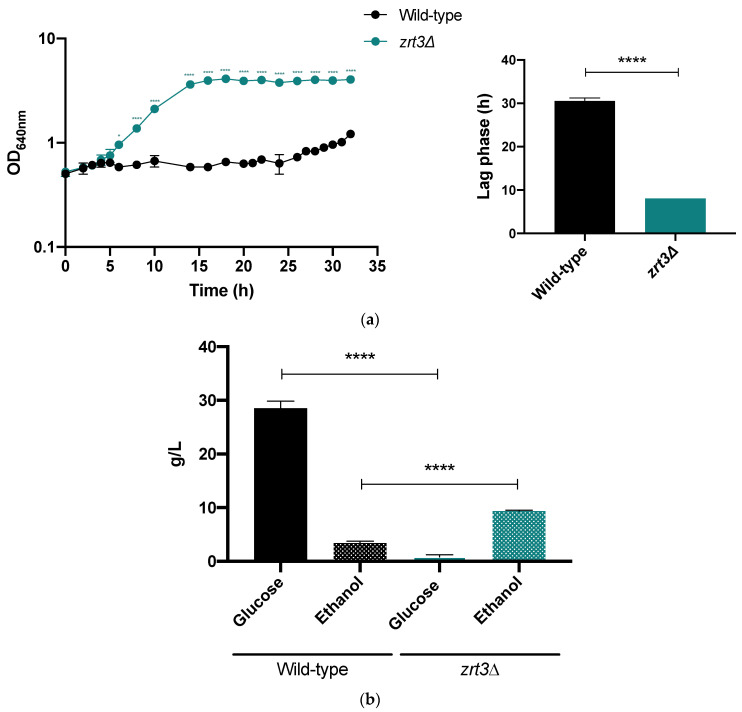
Effect of AA on cell growth and ethanol production of *S. cerevisiae* BY4741 wild-type and *zrt3*Δ strains. (**a**) Cell growth in YEPD medium with 60 mM AA at pH 4.5. Insert: values of the lag phase duration, determined by the time required to double the initial optical density. (**b**) Glucose and ethanol concentrations at the end of the growth experiment (32 h). The values are the mean ±SEM (*n* = 2). Statistical analysis was performed by two-way ANOVA. * *p* < 0.05, **** *p* ˂ 0.0001 comparing the indicated conditions.

Fermentation experiments in YEPD medium in the presence of AA showed a large difference between the performance of the *zrt3*Δ mutant and the wild-type strain regarding both cell growth and ethanol production. Remarkably, while the exponential growth phase of the *zrt3*Δ mutant was initiated 8 h after inoculation, the lag phase of the wild-type strain was much longer (31 h, Figure 5a). This translated into a much higher ethanol production 32 h after inoculation in the mutant strain when compared with the wild-type strain (9.4 g L^−1^ versus 3.4 g L^−1^, respectively) (Figure 5b).

### 3.6. zrt3*Δ* Shows Improved Fermentative Performance in a Lignocellulosic Hydrolysate

**Figure 6:**
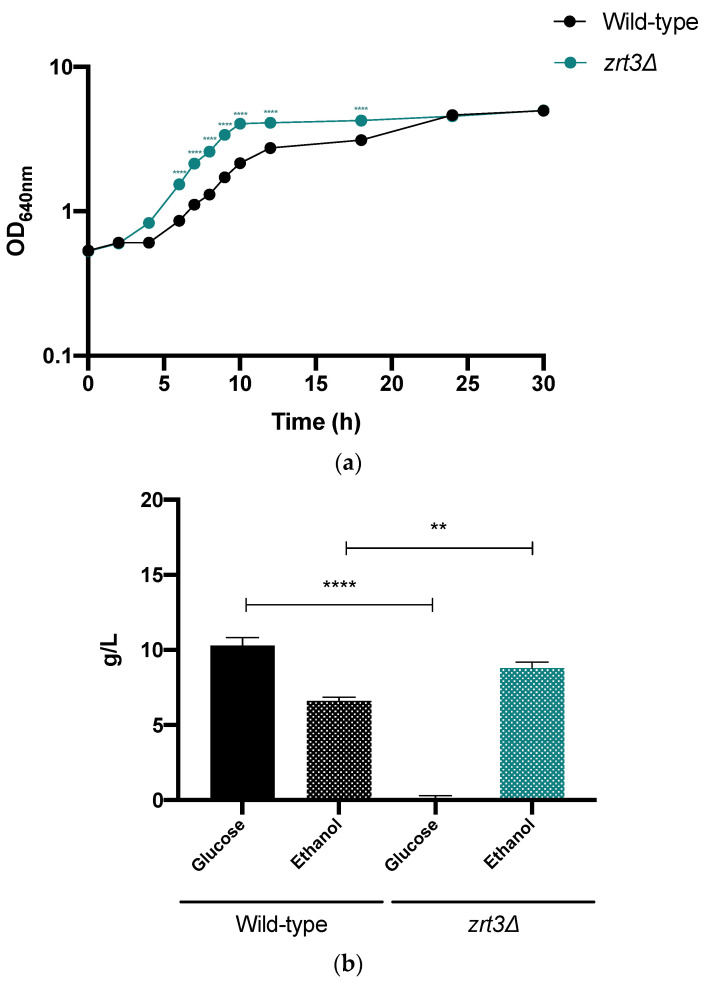
Cell growth, glucose consumption and ethanol production by *S. cerevisiae* BY4741 wild-type and *zrt3*Δ strains on a lignocellulosic hydrolysate. (**a**) Cell growth in a lignocellulosic hydrolysate at pH 4.5 was assessed by measuring optical density along 30 h. (**b**) Glucose and ethanol concentrations were measured 12 h after inoculation, after the *zrt3*Δ strain reached stationary phase. Values are the mean ± SEM (*n* = 3). Statistical analysis was performed by two-way ANOVA. ** *p* ˂ 0.01, **** *p* ˂ 0.0001 in comparison with the indicated conditions.

As reported in Materials and Methods, the lignocellulose biomass used was vine prune residue subjected to autohydrolysis. This pre-treatment resulted in the accumulation of AA (21 mM), formic acid (19 mM), furfural (2 mM) and 5-HMF (2.4 mM). To avoid nutrient limitation, the LH was supplemented with YEPD medium components. Although the OD at stationary phase was identical for both strains 30 h after the inoculation (Figure 6a), the fermentative performance of *zrt3*Δ was better than that of the wild-type strain. Indeed, the lag phase was 5 and 7 h; µ_max_, 0.24 and 0.19 h^−1^ for the *zrt3*Δ strain and wild-type strain, respectively. Importantly, at the end of the exponential phase (12 h), the *zrt3*Δ mutant produced a higher ethanol concentration (Figure 6b).

## 4. Discussion

Among the microorganisms used for industrial bioethanol production, yeasts are the most commonly explored [4], as they can produce high concentrations of ethanol, are able to ferment a wide range of sugars [46] and, importantly, allow low process costs. Cultures are also less prone to contaminations, as they ferment at an acidic pH. *S. cerevisiae* is considered the favorite species for ethanol production because of its high ethanol yield in media with glucose and ethanol tolerance, and is expected to play a central role in multi-waste valorization approaches [47]. However, its main drawback is its incapacity to ferment the xylose that is released during hydrolysis pretreatment of lignocellulosic biomass [2]. *Scheffersomyces stipitis,* previously known as *Pichia stipitis,* is one of the most efficient naturally occurring xylose-fermenting yeasts, showing a high ethanol yield in media with xylose, but it is highly sensitive to ethanol [48,49]. Thus, the construction of xylose-fermenting recombinant *S. cerevisiae* strains has been exploited as a strategy to develop economically viable processes for the production of LE [4,50]. Of note, a synergistic effect of simultaneous expression of the two xylose pathways (xylose reductase/xylitol dehydrogenase and xylose isomerase) with furan detoxification was observed [51]. However, the use of xylose-fermenting strains that are not resistant to AA is still not a competitive strategy for LE production. Indeed, AA is one of the inhibitors derived from lignocellulosic hydrolysis [4] that causes severe stress, limiting yeast ethanol yield and productivity. Therefore, a deeper understanding of the molecular mechanisms underlying resistance to AA will contribute to designing robust strains for sustainable industrial LE production.

This study uncovered that the absence of Zrt3 results in higher cell survival in response to AA, suggesting that Zrt3 is involved in AA-RCD, possibly through its role in the regulation of zinc levels. A recent study identified that the AP-3 adaptor complex is a death-promoting factor during heat-induced cell death and is also involved in vacuole permeabilization. A recent study uncovered that disruption of the AP-3 adaptor complex, which promotes stress-induced cell death by transporting and installing proteins on the vacuolar membrane, confers resistance to AA and hydrogen peroxide [52]. Since Zrt3 is a candidate cargo protein for the AP-3 adaptor complex, those results are consistent with the data obtained in this study linking absence of Zrt3 from the vacuole with a cell death resistance phenotype. 

Previous studies showed that *S. cerevisiae* cells undergoing AA-RCD exhibit accumulation of ROS [22,53]. Cytosolic and mitochondrial superoxide dismutases (Sod1 and Sod2, respectively) are responsible for the conversion of the superoxide anion, generated by the reduction of oxygen, into hydrogen peroxide [54]. In turn, cytosolic and peroxisomal catalases (Cat T and Cat A, respectively) convert hydrogen peroxide into water and oxygen [55]. A well-established set of reactions with these and other antioxidant enzymes allows cells to maintain homeostasis [56]. Sod2, which is particularly required during stationary phase, is involved in mitochondrial oxygen radical detoxification [56]. In the current study, the higher survival of the *zrt3*Δ mutant in response to AA was associated with lower accumulation of both cellular and mitochondrial superoxide anion, in comparison with the wild-type strain. This could be due to higher cytosolic antioxidant defenses, in particular to higher activity of zinc-dependent antioxidant enzymes such as Sod1 and catalase T [57]. In agreement with this hypothesis, the decreased percentage of *zrt3*Δ cells with total cellular accumulation of superoxide anion (DHE-positive) in comparison with the wild-type strain, was higher than the correspondent decrease in the percentage of cells with superoxide anion mitochondria accumulation (Mitosox-positive). Although Sod1 basal activity was identical for both strains, basal activity of catalases was higher in the *zrt3*Δ mutant, which likely results in lower hydrogen peroxide accumulation. In wild-type cells, the basal activity of catalases is probably not sufficient to counteract the accumulation of hydrogen peroxide upon AA stress, which leads to the induction of Sod1, as described for its human ortholog [58]. In turn, the high basal activity of catalases in *zrt3*Δ cells ensures low levels of hydrogen peroxide throughout the course of exposure to AA. This would result in a lack of Sod1 induction and even decreased activity of catalases, whose stimulation depends on hydrogen peroxide levels [59]. This hypothesis is in agreement with the observation that cells pre-adapted to acid stress, where the activity of catalases is enhanced, are resistant to AA-RCD [27]. Although other studies obtained by these authors, as well as by others, are not conclusive about the effect of RCD-inducing concentrations of AA on catalase or superoxide dismutase (SOD) activities, there is a consensus that increased activity of catalases protects cells from AA-RCD [24]. Thus, the results obtained in the current study suggest that the higher activity of catalases endows the *zrt3*Δ mutant with greater resistance to AA, in accordance with a major role of hydrogen peroxide detoxification in the prevention of AA-RCD [21]. 

AA is also known to induce vacuolar membrane permeabilization (VMP), along with the release of Pep4 to the cytosol [25]. This vacuolar protease plays a role in mitochondrial degradation during AA-RCD and, depending on the strain background, can play a protective or an executioner role [25,60]. In fact, BY4741 yeast cells lacking Pep4 become more resistant to AA, suggesting an executioner role in this strain background [26]. Interestingly, both Pep4 release and VMP were delayed in the *zrt3*Δ mutant, consistent with its increased cell survival. Moreover, wild-type cells exhibited significant vacuolar alkalinization in response to AA, while the acidic pH is maintained in *zrt3*Δ cells. These data support the interpretation that the increased survival of the *zrt3*Δ mutant to AA is associated with a delayed perturbation in vacuolar function, including a later VMP and Pep4 release.

It was previously found that zinc protects yeast cells from oxidative damage caused by AA [61]. Another study with *S. cerevisiae* demonstrated that zinc modulates cellular amino acid metabolism and redox balance, particularly the biosynthesis of alanine and glutathione, to exert its antioxidant effect [28]. In agreement, supplementation with ZnSO_4_ was shown here to protect wild-type cells from AA-RCD. However, this protective effect was not associated with a significant decrease in superoxide anion levels. On the other hand, the limitation of zinc rendered cells more sensitive to AA-RCD, and led to increased superoxide anion levels. While an effect of zinc-chelator EDTA on the homeostasis of other cations and cell processes cannot be ruled out, these results are in accordance with previous reports that *S. cerevisiae* shows increased ROS levels under zinc-limiting conditions [61]. Taken together, these data suggest that zinc attenuates the cytotoxic effects of AA, but not by minimizing the accumulation of superoxide anion. 

Although the wild-type strain displays increased AA resistance when supplemented with zinc, and this supplementation is known to improve fermentation efficiency, above certain concentrations it becomes cytotoxic [28,30,31]. Indeed, besides being associated with additional costs, zinc supplementation can affect cell function and ultimately compromise ethanol productivity, if not carefully handled. Thus, the exploitation of the *zrt3*Δ mutant in LE production emerges as a more advantageous alternative, as it avoids supplementation of LH with zinc.

To characterize the performance of *zrt3*Δ cells under conditions more similar to LE production, their fermentation profile was monitored under AA stress conditions. It is well established that AA originates from acetyl-groups of hemicellulose during the pre-treatment that lignocellulosic biomass undergoes to release fermentable sugars [17,62]. Depending on the biomass and hydrolytic process used, AA can be found in concentrations between 8 and 192 mM (0.5 and 11.5 g/L) [63]. Here, the *zrt3*Δ mutant stood out when compared with the wild-type regarding both cell growth and fermentative performance under 60 mM (3.6 g/L) AA stress, with a 2.6-fold improvement in ethanol productivity. These results are in agreement with previous reports showing that a xylose-recombinant *S. cerevisiae* strain subjected to 30 mM AA stress produces more ethanol in the presence of 180 μM ZnSO_4_ [31]. The improved growth of the *zrt3*Δ strain, and consequently its fermentative performance, may rely on the fact that this mutation causes upregulation of zinc transport across the plasma membrane to compensate for zinc scarcity, resulting in a richer zinc pool. Accordingly, under zinc-replete conditions, there is a higher zinc accumulation in *zrt3*Δ mutants than in the wild-type strain [36]. Although these results were encouraging, the presence of many different inhibitors in LH has been reported to have a synergistic effect on yeast growth inhibition [64,65]. Therefore, the fermentation profile of wild-type and *zrt3*Δ mutant cells in vine prune residue autohydrolysis liquors containing AA (21 mM), formic acid (FA, 19 mM), furfural (F, 2 mM) and 5-hydroxymethylfurfural (HMF, 2.4 mM) was compared. The *zrt3*Δ strain still displayed increased fermentative capacity and a 1.3-fold improvement in ethanol productivity in comparison with the wild-type strain. Despite the observed difference in productivity, ethanol yield was similar for the two strains, as for both cases 1 g of glucose was converted to 0.23 g of ethanol, which corresponds to 45% of the theoretical maximum yield. Although the concentration of each inhibitor per se was relatively low in the LH, the observed cell growth inhibition and ethanol yield reduction are likely a result of their combined inhibitory effects. Whether the absence of Zrt3 also protects against FA, F and HMF alone or in combination with AA is currently being addressed.

## 5. Conclusions and Future Perspectives

Acetic acid is the main inhibitor of fermentation of lignocellulosic hydrolysates. Given its influence in the fermentative process, and consequently in the production of ethanol, it is increasingly important to find strategies to overcome this limitation. In this study, we aimed to clarify the role of the zinc transporter Zrt3 in the mechanisms underlying acetic acid-induced RCD in yeast, towards the improvement of LE production. Taken together, the results indicate that the *zrt3*Δ mutant displays higher resistance to cell death induced by acetic acid and an improved fermentation profile, particularly a higher level of ethanol productivity, in comparison with the wild-type strain. These data validate the potential of exploiting the *zrt3*Δ mutant in the bioethanol industry as a much more practical and cost-effective alternative to medium supplementation with zinc that had been proposed to mitigate the effects of the LH-derived inhibitor AA. Moreover, the results confirm the hypothesis that the zinc vacuolar transporter Zrt3 is involved in vacuole-mediated AA-RCD.

In the near future, the next step will be the validation of the applicability of our findings in the industrial production of bioethanol from LH through the deletion of *ZRT3* in a *S. cerevisiae* xylose-utilizing strain. Furthermore, studies should be directed towards determining the sustainability of using this mutant strain. The use of methodologies such as life cycle assessment (LCA) and exergy, together with environmental and economic analyses (e.g., exergoenvironmental and exergoeconomic analyses, respectively), will allow for an adequate assessment of the degree of sustainability of the proposed solution for LE production [66,67].

## Figures and Tables

**Table 1:** Recent studies on the application of engineered *Saccharomyces cerevisiae* strains towards improved tolerance to inhibitors in lignocellulosic hydrolysates. Adapted from [6], with permission of Springer Nature, 2022.

*S. cerevisiae* Strain	Modifications	Lignocellulosic Hydrolysate Composition (g/L)	Outcomes	References
D452-2 (MATα, leu2, his3, ura3, and can1)	Expression of the *adhE* and *ACS* gene from *E. coli* and a *Salmonella* mutant, respectively; xylose consumption: multiple copies of *XYL1*, *XYL2*, and *XYL3* (genes constituting the xylose-assimilating pathway), and deletion of *PHO13* and *ALD6*	*Miscanthus* hydrolysate (20 glucose, 50 xylose, 10 acetic acid, 1 HMF, and 2 furfural)	Acetic acid consumption and faster xylose consumption; higher ethanol production and lower glycerol and xylitol production	[8]
BY4741 (MATa, his3Δ1, leu2Δ0, met15Δ0, ura3Δ0)	Overexpression of *YAP1*, *STB5*, *WAR1*, *PDR8*, *CAT8*, *PUT3*, and *GZF3*, separately	Sugarcane bagasse hydrolysate (0.89 furfural, 0.11 HMF,1.4 acetic acid, 0.03 formic acid and 0.05 levulinic acid); spruce hydrolysate (0.36 furfural, 0.03 HMF, 0.72 acetic acid, 0.27 formic acid, and 0.12 levulinic acid)	Increased relative growth rates	[9]
D452-2 (MATα, leu2, his3, ura3, and can1)	Overexpression of *SPE3* and deletion of *TPO1* and *OAZ1*	Corn stover hydrolysate (3.3 acetic acid, 0.8 HMF, and 0.4 furfural)	Improved ethanol productivity	[10]
Haploid derivative of ATCC 4124 strain	Expression of the *adhE* gene from *E. coli*; xylose consumption: expression of *XYL1*, *XYL2*, and *XYL3* from *Scheffersomyces stipitis* and deletion of *PHO13* and *ALD6*	*Miscanthus* hydrolysate (20 glucose, 55 xylose, 10 acetic acid, 1 HMF, and 2 furfural)	Higher ethanol productivity and lower by-product yield	[11]
D452-2 (MATα, leu2, his3, ura3, and can1)	Overexpression of *GRE2*; xylose consumption: multiple copies of *XYL1*, *XYL2*, and *XYL3*, and knockout *ALD6*	*Miscanthus* hydrolysate (20 glucose, 50 xylose, 10 acetic acid, 1 HMF, and 2 furfural)	Greater robustness towards toxic hydrolysate; increased xylose consumption rate, inhibitor tolerance and ethanol production	[12]
BY4742 (MATa, his3Δ1, leu2Δ0, lys2Δ0, ura3Δ0)	Overexpression of *TRX1*	Diluted bagasse hydrolysate (44 glucose, 5.8 xylose, 4.1 acetic acid, 0.6 furfural, and 0.2 HMF)	Larger ethanol production in titter, yield, and productivity; increased levels of protectant metabolites (trehalose, fatty acids, GABA and putrescine)	[13]
PE-2 (NCYC 3233)	Overexpression of *HAA1* and/or *PRS3*; Xylose consumption: expression of *XYL1* and *XYL2* from *Schef. stipitis*, overexpression of *XKS* and *TAL1*, and deletion of *GRE3*	Paulownia hydrolysate (5.84 acetic acid, 1.96 furfural, and 0.72 HMF)	Improved yeast adaptation to non-detoxified hydrolysate with high acetic acid content; increased yeast cell wall robustness under acetic acid stress situations	[14]
XUSAE57	Overexpression of *TAL1* and *XKS1* and deletion of *GRE3* and *PHO13*	Sugarcane bagasse hydrolysate(34 glucose, 32 xylose, 3.1 acetic acid, 0.7 phenolics)	Improved xylose utilization and higher ethanol yield	[15,16]

## Data Availability

Not applicable.

## Abbreviation

AA	Acetic acid
CAT	Catalase
CDCFDA	5-(and -6)-carboxy-2′, 7′-dichlorofluorescein diacetate
c.f.u.	Colony Forming Units
CMAC	7-amino-4-chloromethylcoumarin
DIC	Differential Interference Contrast
DHE	Dihydroethidium
DMSO	Dimethyl sulfoxide
F	Furfural
FA	Formic acid
GHG	Greenhouse gases
HMF	Hydroxymethylfurfural
LE	Lignocellulosic ethanol
LH	Lignocellulosic hydrolysates
NBT	Nitro Blue Tetrazolium
RCD	Regulated cell death
ROS	Reactive oxygen species
*S. cerevisiae*	*Saccharomyces cerevisiae*
*Schef. stipitis*	*Scheffersomyces stipitis*
SOD	Superoxide dismutase
VMP	Vacuolar membrane permeabilization
Vpr	Vine pruning residue
YEPD	Yeast Extract Peptone Dextrose
